# Hip and knee arthroplasty: quo vadis?

**DOI:** 10.1186/s13756-015-0060-9

**Published:** 2015-05-15

**Authors:** Jeffery Ho, Jacques F Meis, Marrigje Nabuurs-Franssen, Andreas Voss

**Affiliations:** School of Nursing, The Hong Kong Polytechnic University, Kowloon, Hong Kong; Department of Medical Microbiology and Infectious Diseases, Canisius-Wilhelmina Hospital, Nijmegen, The Netherlands; Department of Medical Microbiology, Radboud university medical centre, Nijmegen, The Netherlands; Department of Anesthesia and Intensive Care, Prince of Wales Hospital, The Chinese University of Hong Kong, Shatin, Hong Kong

**Keywords:** Surgical infection, Hip and knee arthroplasty, The Netherlands

## Abstract

Despite of the steady decrease of surgical site infection (SSI) over the last two decades, the incidence of SSI after hip and knee arthroplasty has recently surged. This may be explained by technical changes that may result in an increased risk of SSI, such as the broad implementation of fast track programs, and/or early interventions on suspected SSI. By definition, early intervention may lead to a higher SSI score, even in the absence of a true SSI. In any case, the reverse trend of SSI warrants further investigations.

Over the last two decades it has been shown, that various infection control measures are able to decrease the rate of surgical site infection (SSI) for total hip and knee arthroplasty (THA/TKA) [1-3]. In the Netherlands, the incidence of SSI after THA/TKA fell from 3% to 1.5% during the early years of the present millennium [4] and seemed to have levelled out at 1-2% during the last years, according to the Dutch national surveillance system for healthcare-associated infections (PREZIES). Most recently, and despite the above mentioned consistent national trend, the Dutch Health Care Inspectorate (IGZ) received reports from a significant number of orthopaedic surgery departments, that their rates of SSI after THA/TKA rose to more than 2%, which is presently seen as the acceptable upper limit of SSI-rate for this type of surgery. While an incidental, temporary increase in any kind of SSI rate may be explained by local circumstances, the reverse trend in national incidence rates for multiple centres demands further investigations.

What were the changes in the pre-, peri-, and post-operative procedures with regard to THA and TKA? Most importantly, which of those changes were implemented on a (close to) national level, in order to achieve the observed effect? We believe that two major practice changes might be responsible for the recent changes, or at least a major part of it: “fast-track” and “early intervention”.

The recent introduction of fast-track programs in THA and TKA has attracted increasing appreciation in promoting functional restoration, minimising co-morbidities and shortening the duration of hospital stay [5,6]. However, the question remains: has the implementation of apparently successful fast-track orthopaedic surgery been compromised by higher risk of infections? The length of stay for fast-track cohorts can be as short as two days as compared with the conventional (up to) seven days [5]. Undoubtedly, this shortened hospitalisation period can lighten the healthcare burden and increase patient satisfaction. However, the early mobilisation as well as the changes in the number of drains that are placed, or better not placed, may result in suture dehiscence and/or increased wound leakage which by consequence, may increase the risk of SSI.

In addition, fall injury appears to be rather common during the first week after fast track THA/ TKA, attributable to 25% of the fall incidences [7], which might otherwise be preventable if the patient remained hospitalised. Therefore, early discharge from fast-track arthroplasty may increase risk for fall injury, which may thereby disrupt newly closed surgical wounds, contributing to subsequent infection. In a recent Danish case–control study, patients undergoing fast-track THA/ TKA were found to have 30% increased risk for infection, although the origins of the infection foci were not comprehensively described [6]. This study compared 17,284 fast track arthroplasties with 61,814 conventional arthroplasties in 2005 – 2011. Whilst the fast track cohort had a 30% reduced risk for thromboembolic event and similar risk for re-operation and mortality, this group exhibited a significantly higher risk of re-admission due to infection (RR = 1.3, 95% CI: 1.1 – 1.6). Although the risk ratio appeared to be small, the authors noted that a number of hospitals included in the national cohort had, to some extent, implemented fast track program, albeit not as systematically and/ or consistently as fast track participanting institutes.

SSI is a consequence of multifactorial risk factors, including pre-existing medical conditions and demographics [8]. Whether fast-track programs for THA/TKA poses a higher risk for SSI needs to be clarified. Future investigations should certainly look into components of fast-track surgery, such as changes in the number and placement of drains and early mobilisation and try to evaluate their influence against the changes with regard to the increasing presence of other risk-factors, such as obesity, diabetes mellitus, and others.

In addition, the recent surge of SSI incidence may – in part - be explained by early interventions on suspected cases of SSI after arthroplasty [9]. When SSI is suspected or persistent wound leakage is present, orthopaedic surgeons can either wait and see, with or without antibiotic coverage, or explore the surgical wound. This is frequently accompanied by placing a drain in an attempt to remove any wound leakage. According to the current surveillance guidelines (PREZIES), SSI is defined as the presence of signs of local inflammation detected within one year after arthroplasties, accompanying with purulent drainage from incision, wound dishescence or wound opened by surgeons. Therefore, the placement of a drain in the surgical wound has already fulfilled one of the criteria of defining SSI regardless of the bacterial culture results. This may lead to a “pseudo” increase in SSI, since we believe that the surgeons trend to explore the wound at an earlier time, than they used to. While early interventions may be crucial in the final outcome of the patient (implant retention), they may negatively influence the SSI-rate. Consequently, we suggest including additional outcome indicators such as implant retention after e.g. two years to exclude “pseudo” SSI.

In summary, whether the recent increase in SSI incidence in multiple Dutch hospitals is a consequence of introduction of novel techniques such as “fast-tract programs” or an apparent “pseudo” increase in SSI incidence as a result of a change of treatment strategies such as “early interventions” remains to be elucidated.

## References

[1] Matt M, Clohisy J, Warren D, Hopkins-Broyles D, McMullen K (2005). Decreased surgical site infection (SSI) rates for hip and knee arthroplasty following multiple infection control interventions. Am J Infect Control.

[2] Hadley S, Immerman I, Hutzler L, Slover J, Bosco J (2010). *Staphylococcus aureus* decolonization protocol decreases surgical site infections for total joint replacement. Arthritis.

[3] Ponce B, Raines BT, Reed RD, Vick C, Richman J, Hawn M (2014). Surgical site infection after arthroplasty: comparative effectiveness of prophylactic antibiotics: do surgical care improvement project guidelines need to be updated?. J Bone Joint Surg Am.

[4] Mannien J, van den Hof S, Muilwijk J, van den Broek PJ, van Benthem B, Wille JC (2008). Trends in the incidence of surgical site infection in the Netherlands. Infect Control Hosp Epidemiol.

[5] Krenk L, Jennum P, Kehlet H (2013). Activity, sleep and cognition after fast-track hip or knee arthroplasty. J Arthroplasty.

[6] Glassou EN, Pedersen AB, Hansen TB (2014). Risk of re-admission, reoperation, and mortality within 90 days of total hip and knee arthroplasty in fast-track departments in Denmark from 2005 to 2011. Acta Orthopaedica.

[7] Jorgensen CC, Kehlet H (2013). Fall-related admissions after fast-track total hip and knee arthroplasty – cause of concern or consequence of success?. Clin Interv Aging.

[8] Reina N, Delanary C, Chiron P, Ramdane N, Hamadouche M (2013). Societe francaise de chirurgie orthopedique et traumatologique. Orrthop Traumatol Surg Res.

[9] Geurts JAP, Janssen DMC, Kessels AGH, Walenkamp GHIM (2013). Good results in postoperative and hematogenous deep infections of 89 stable total hip and knee replacements with retention of prosthesis and local antibiotics. Acta Orthopaedica.

